# Phase 1 study of Z-endoxifen in patients with advanced gynecologic, desmoid, and hormone receptor-positive solid tumors

**DOI:** 10.18632/oncotarget.27887

**Published:** 2021-02-16

**Authors:** Naoko Takebe, Geraldine O'Sullivan Coyne, Shivaani Kummar, Jerry Collins, Joel M. Reid, Richard Piekarz, Nancy Moore, Lamin Juwara, Barry C. Johnson, Rachel Bishop, Frank I. Lin, Esther Mena, Peter L. Choyke, M. Liza Lindenberg, Larry V. Rubinstein, Cecilia Monge Bonilla, Matthew P. Goetz, Matthew M. Ames, Renee M. McGovern, Howard Streicher, Joseph M. Covey, James H. Doroshow, Alice P. Chen

**Affiliations:** ^1^Division of Cancer Treatment and Diagnosis, National Cancer Institute, Bethesda, MD 20892, USA; ^2^Department of Oncology, Mayo Clinic, Rochester, MN 55905, USA; ^3^Leidos Biomedical Research, Inc., Frederick National Laboratory for Cancer Research, Frederick, MD 21702, USA; ^4^Consult Services Section, National Eye Institute, Bethesda, MD 20892, USA; ^5^Molecular Imaging Program, National Cancer Institute, Bethesda, MD 20892, USA; ^6^Biometric Research Program, National Cancer Institute, Bethesda, MD 20892, USA; ^7^Center for Cancer Research, National Cancer Institute, Bethesda, MD 20892, USA; ^8^Division of Hematology and Medical Oncology, Knight Cancer Institute, Oregon Health & Science University, Portland, OR 97239, USA

**Keywords:** Z-endoxifen, phase 1, tamoxifen, pharmacokinetics

## Abstract

Background: Differential responses to tamoxifen may be due to inter-patient variability in tamoxifen metabolism into pharmacologically active Z-endoxifen. Z-endoxifen administration was anticipated to bypass these variations, increasing active drug levels, and potentially benefitting patients responding sub-optimally to tamoxifen.

Materials and Methods: Patients with treatment-refractory gynecologic malignancies, desmoid tumors, or hormone receptor-positive solid tumors took oral Z-endoxifen daily with a 3+3 phase 1 dose escalation format over 8 dose levels (DLs). Safety, pharmacokinetics/pharmacodynamics, and clinical outcomes were evaluated.

Results: Thirty-four of 40 patients were evaluable. No maximum tolerated dose was established. DL8, 360 mg/day, was used for the expansion phase and is higher than doses administered in any previous study; it also yielded higher plasma Z-endoxifen concentrations. Three patients had partial responses and 8 had prolonged stable disease (≥ 6 cycles); 44.4% (8/18) of patients at dose levels 6–8 achieved one of these outcomes. Six patients who progressed after tamoxifen therapy experienced partial response or stable disease for ≥ 6 cycles with Z-endoxifen; one with desmoid tumor remains on study after 62 cycles (nearly 5 years).

Conclusions: Evidence of antitumor activity and prolonged stable disease are achieved with Z-endoxifen despite prior tamoxifen therapy, supporting further study of Z-endoxifen, particularly in patients with desmoid tumors.

## INTRODUCTION

Tamoxifen is a member of the selective estrogen receptor modulator (SERM) drug family and is approved by the FDA for the treatment of patients with estrogen receptor-positive (ER+) metastatic breast cancer, for adjuvant therapy of high-risk ER+/progesterone receptor-positive (PR+) breast cancer, and for chemoprevention in women at high risk of developing breast cancer [1, 2]. Tamoxifen binds to the ligand-binding domain of the ER, blocking the binding of estrogens and the transcriptional activation of estrogen response genes, thereby inhibiting tumor growth [3]. However, only about 50% of women with metastatic ER+ breast cancer who receive treatment with tamoxifen derive benefit, and trials have yielded mixed results regarding the clinical benefit of tamoxifen based on dose or serum concentration [4–7].

Despite its lengthy history of clinical use, factors contributing to tamoxifen metabolism are not clearly understood. One established fact is that tamoxifen itself is a weak anti-estrogenic agent [8, 9]. Tamoxifen is metabolized by hepatic cytochromes P450 (CYPs) via two distinct pathways [6]. CYP3A4/5 is the major CYP isoform responsible for the conversion of a large percentage of tamoxifen into N-desmethyltamoxifen (NDM-tamoxifen). CYP2D6 is the only enzyme responsible for converting NDM-tamoxifen into endoxifen [10]. CYP2D6 also converts a small percentage of tamoxifen into 4-hydroxy-tamoxifen and then into endoxifen [6]. Endoxifen and 4-hydroxy-tamoxifen have similar binding affinities for ERα and ERβ, which are approximately 100-fold higher than those of tamoxifen or NDM-tamoxifen, but endoxifen plasma concentrations following tamoxifen administration are 5- to 20-fold higher than 4-hydroxy-tamoxifen. Endoxifen and 4-hydroxy-tamoxifen are both present as two isomers, Z- and E-. The Z-isomers of each compound have similar anti-estrogenic activity and are more active than the E-isomers [11–13]. Z-endoxifen is therefore thought to account for a substantial proportion of the clinical activity of tamoxifen [2, 8, 14, 15].

Studies have evaluated the effect of increasing tamoxifen doses in humans, thereby increasing the circulating concentration of pharmacologically active Z-endoxifen [16–20]. More than 100 polymorphisms in CYP2D6 have been reported and linked to variations in endoxifen levels following administration of tamoxifen [21, 22]. Multiple other factors, including age [23], body mass index (BMI) [24], gender [25], and polypharmacy [15, 26] contribute to how patients metabolize tamoxifen into endoxifen. All of these factors contribute to the variability of endoxifen pharmacokinetics. Among patients who receive tamoxifen, levels of endoxifen are lower in poor metabolizers (decreased CYP2D6 activity), a finding that appears to correlate with significantly reduced time to tumor recurrence in these patients compared to those with greater CYP2D6 metabolism following treatment with adjuvant tamoxifen [10].

Our current trial examined the safety and tolerability of Z-endoxifen in patients with gynecologic tumors, desmoid tumors, hormone receptor-positive (HR+) breast cancer, or other HR+ solid tumors at oral doses up to 360 mg daily. Blood and urine samples were collected to evaluate the pharmacokinetic profile of Z-endoxifen. In a subset of patients, ^18^F-FES (16 alpha-[18F]-fluoro-17 beta-estradiol) PET/CT imaging studies confirmed ER binding of Z-endoxifen. Administration of Z-endoxifen was well tolerated, but resulted in an adverse event profile distinct from that observed with tamoxifen at higher plasma levels of the active metabolite.

## RESULTS

### Patient demographics

Forty patients with advanced, refractory gynecologic tumors, desmoid tumors, hormone receptor-positive breast cancer, or other hormone receptor-positive cancers were enrolled on the study between March 2011 and September 2017 (Table 1). Nineteen of these patients had received prior treatment with tamoxifen and/or an aromatase inhibitor. All 9 patients with breast cancer had received prior aromatase inhibitor therapy; 7 of these patients also received prior tamoxifen therapy (Supplementary Table 1).

**Table 1:** Summary of patient demographics and clinical histories

Patient Characteristics	*n*	%
Number of patients enrolled:	40	
Number of patients evaluable:	34	85
Median age, years:	60	
Age range, years:	21–80	
ECOG Performance status:		
0	6	15
1	34	85
Sex:		
Male	4	10
Female	36	90
Diagnosis:		
Ovarian cancer	10	25
Breast cancer	9	22.5
Endometrial cancer	8	20
Desmoid fibromatosis	6	15
Fallopian tube cancer	3	7.5
Granulosa cell ovarian	2	5
Cervical cancer	1	2.5
Uterine leiomyosarcoma	1	2.5
Hormone receptor status		
ER+PR+	15	37.5
ER+PR-	5	12.5
ER-PR+	1	2.5
ER-PR-	1	2.5
Undetermined	18	45
Prior therapies:		
Prior hormone treatment	19	47.5
No prior hormone treatment	21	52.5

### Clinical pharmacology

**Figure 1:**
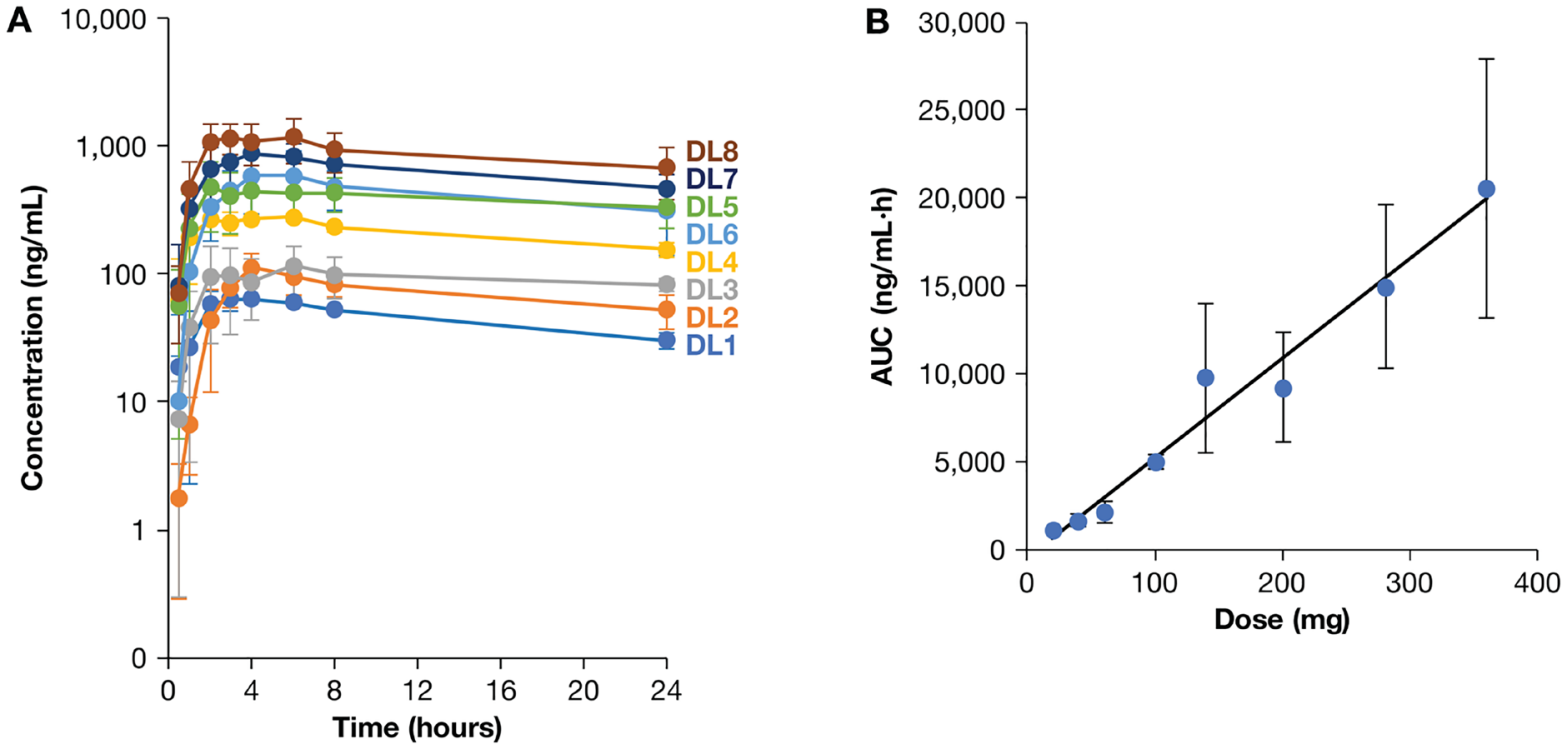
Day 1 PK data by dose level. (**A**) Mean Z-endoxifen plasma concentrations for patients at all dose levels. Data points represent means +/– standard deviations for all patients at each dose level at indicated time points on day 1, except for patients 35 and 36 who received the free base form of Z-endoxifen on that day. (**B**) Drug exposure on day 1 at all dose levels. AUC_(0-24h)_ increased linearly with dose. Data points indicate mean plus/minus standard deviations for all patients at each dose level, again excluding patients 35 and 36.

Mean plasma Z-endoxifen concentrations for each dose level on day 1 of cycle 1 are presented in Figure 1A. The results of non-compartmental pharmacokinetic analysis for all patients, except patients 35 and 36, are provided in Supplementary Table 2. Area under the concentration-time curve for 24 hours (AUC_(0–24h)_) values demonstrate a linear increase with dose (Figure 1B). The elimination half-life (t_½_) was 30.6–55.9 hours. Day 28 PK data are available for patients at DL1-6, as the 2 μM C_max_ goal was achieved at DL6 (2.86 μM); the mean C_24 h_ value on Day 28 at DL6 was more than 180-fold higher than the 5.9 ng/mL threshold previously associated with clinical benefit for patients receiving tamoxifen [21]. Plasma concentrations of Z-endoxifen 24 hours after the first dose in the current study ranged from 67 nM at DL1 to 1810 nM at DL8 (calculated from the C24h values reported in units of ng/mL in Supplementary Table 2). At day 28 of this study, the average DL1 (20 mg/day) plasma concentration of Z-endoxifen was 353 nM, a 4- to 18-fold increase over 4-month plasma concentrations reported with a 20 mg daily dose of tamoxifen by Jin et al. [26]. Plasma concentrations on day 1 at DL8 (676 ng/mL with a 360 mg dose) were approximately two-fold higher than in the first-in-human trial of Z-endoxifen in 38 patients with ER+ metastatic breast cancer (333 ng/mL with a 160 mg dose) [27]. Patients 35 and 36 received free base Z-endoxifen on day 1 to compare PK values with the HCl salt. Day 1 (free base) PK profiles in these two patients were highly variable and bracketed the mean AUC for other patients receiving the HCl salt at this dose level (Supplementary Table 3). Day 2 (HCl salt) exposures for patients 35 and 36 were higher than those on day 1, but the data are too limited to conclude any formulation advantage. Plasma E-endoxifen concentrations were < 2% of Z-endoxifen levels. Previous studies demonstrated that endoxifen concentration is lower in the urine than in bile [28] and tamoxifen clearance is driven by the liver [6]. Consistent with these previous reports, very low amounts (< 0.26% of dose) of Z-endoxifen were excreted in the urine (data not shown). These data indicate that Z-endoxifen does not require dose adjustment based on a patient’s renal function and will drive future work with Z-endoxifen.

**Figure 2:**
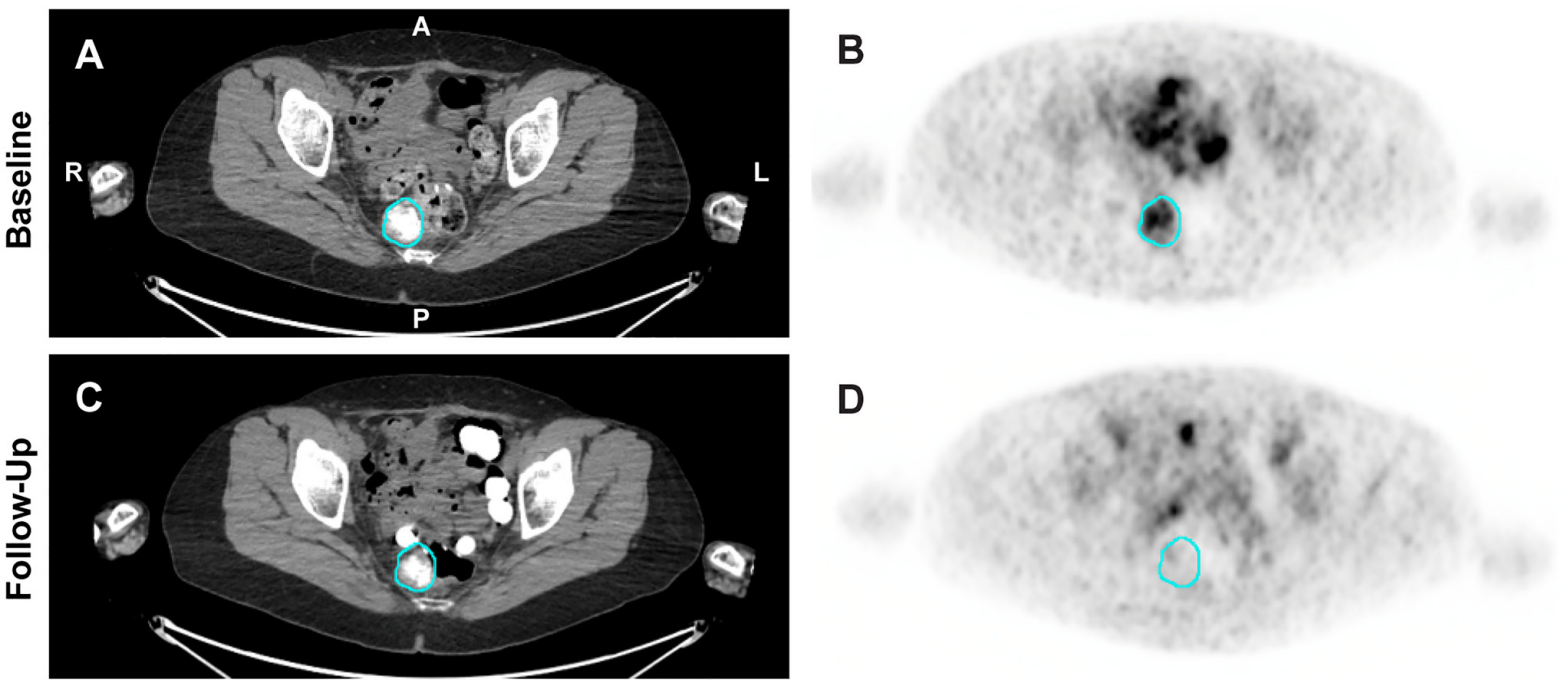
^18^F-FES PET/CT images from patient 40 with serous ovarian cancer. Imaging was performed prior to treatment ((**A**) [CT] and (**B**) [PET]) and after 3 days of treatment with Z-endoxifen at DL8 ((**C**) [CT] and (**D**) [PET]). The blue circles indicate the location of a pre-sacral soft tissue lesion.

The pharmacodynamic effect of Z-endoxifen, blocking estrogen receptor binding, was assessed by ^18^F-FES PET/CT imaging. ER+ tumors show significant uptake of ^18^F-FES on PET/CT scans due to high affinity binding of the tracer to the ERα; this imaging approach has previously been shown to be a pharmacodynamic marker for Z-endoxifen treatment [29]. Twenty patients in this trial agreed to be screened by ^18^F-FES PET/CT imaging to assess ER status. Ten patients who were positive prior to treatment were re-imaged from 1 to 5 days after treatment initiation. We present here images taken after 3 days of treatment from a patient with serous ovarian cancer whose imaging has not previously been reported (patient 40, DL8, Figure 2). This patient had been treated with multiple regimens prior to enrolling in this trial, including a combination of tamoxifen, anastrozole, and letrozole for over 3 years. This patient developed a bowel obstruction during cycle 1 attributed to her disease and chose to come off study. Other images from patients in this trial have been previously reported [29].

### Safety

Z-endoxifen was generally well tolerated (Supplementary Table 4). The most frequent study-related adverse events were grades 2 and 3 lymphopenia (*n* = 11) and anemia (*n* = 10). Three grade 4 adverse events were observed that were potentially related to the study agent (one each of colonic perforation, hypophosphatemia, and a thromboembolic event). One patient on DL2 (40 mg/day, patient 4) developed a grade 4 pulmonary embolism that was considered a DLT. Three additional patients were enrolled at DL2; no additional grade 3 or 4 adverse events were observed at this dose level. A second DLT, grade 3 ALT elevation, occurred at DL7 (280 mg/day, patient 22). Four additional patients were enrolled at DL7, with one patient refusing further treatment. No additional DLTs were observed at DL7, and escalation continued. A patient with fallopian tube carcinoma treated at DL8 (360 mg) with extensive abdominal disease extending to the pelvic wall experienced a grade 4 colonic perforation at the end of cycle 1. While drug attribution could not be ruled out due to the timing of administration relative to the event, the patient’s disease was determined to be the most likely cause. Further escalation was suspended per protocol due to the pill burden and exceeding the defined target plasma level of Z-endoxifen. DL8 was established as the dose for the expansion phase; additional patients were enrolled to a total of 12 patients at DL8.

### Clinical outcomes

**Figure 3:**
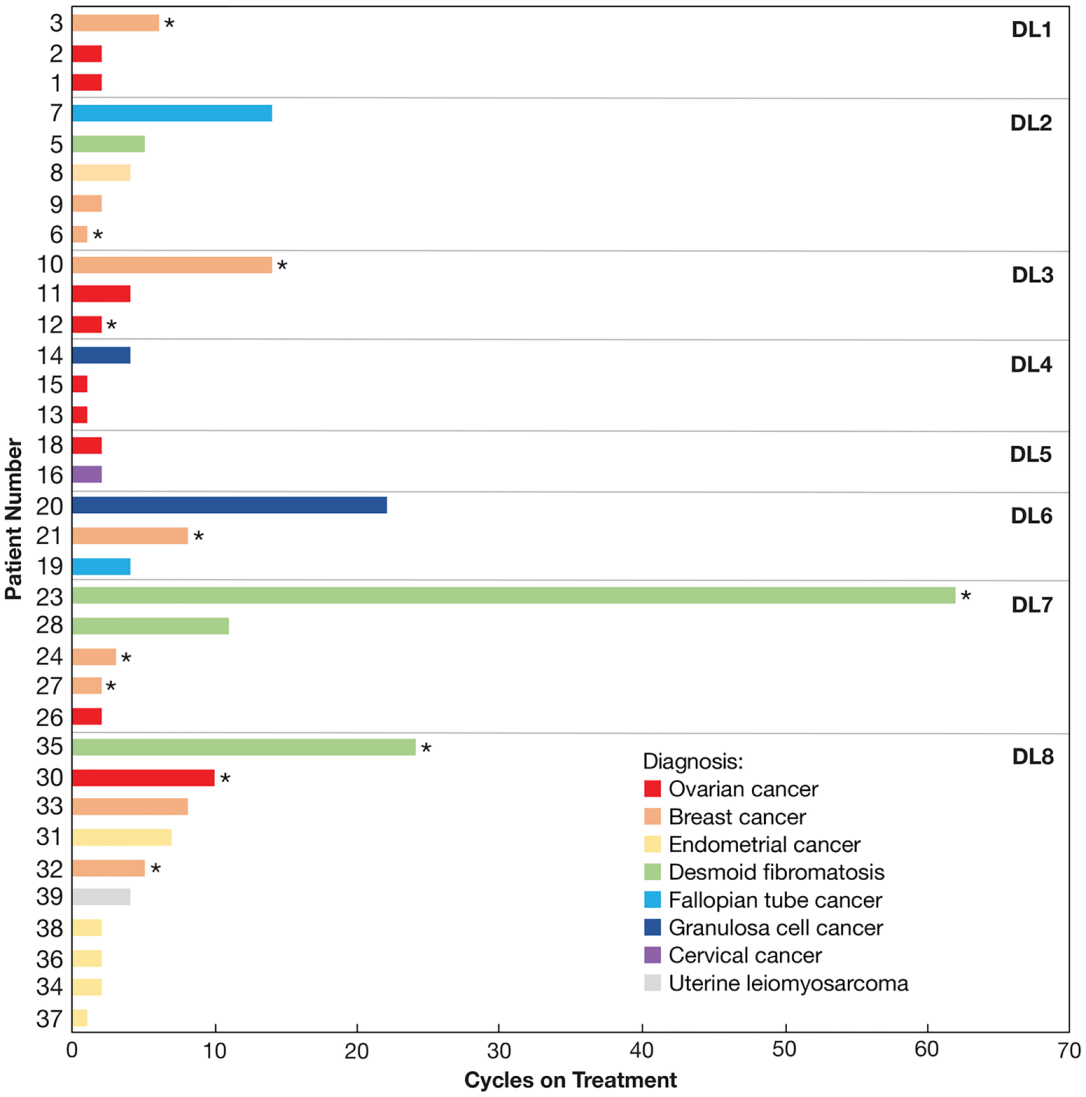
Number of cycles completed by each evaluable patient. Colors indicate the diagnosis of each patient as indicated. Asterisks indicate patients who had previously progressed on tamoxifen therapy.

Thirty-four patients were evaluated for clinical response to Z-endoxifen treatment. These patients remained on study for 1–62 treatment cycles (average = 7.2 cycles; median = 4 cycles) (Figure 3). At DL1 to DL5, 18.8% of patients (3/16 patients, 2 patients with breast cancer and 1 with fallopian tube cancer) experienced a partial response or stable disease for ≥ 6 cycles; these outcomes were observed in 44.4% of patients treated at DL6 to DL8 (8/18 patients, 3 patients with desmoid tumors, 2 with breast cancer, 2 with ovarian cancer, and 1 with endometrial cancer).

### Patients with desmoid tumors

Four patients with desmoid fibromatosis were evaluable for clinical outcome. Patient 23, who had previously progressed on tamoxifen and γ-secretase inhibitor therapy, had a partial response. This patient continues on study (62+ cycles) at time of data cut off and reports improved pain levels such that he no longer requires narcotics. Patient 28, who previously progressed on multiple therapies including sorafenib, and patient 35, who previously progressed on tamoxifen, had prolonged disease stabilization (11 and 24 cycles, respectively). Patient 35 (DL8) also reported a subjective response characterized by softening of the tumor allowing for bending of the knee joint starting around cycle 10. Patient 28 (DL7) died of an undetermined cause after 11 cycles.

### Patients with gynecologic tumors

Twenty patients with gynecologic tumors were evaluable. These patients’ diagnoses included ovarian (*n* = 9), endometrial (*n* = 6), fallopian tube (*n* = 2), granulosa cell ovarian (*n* = 2), and cervical cancer (*n* = 1). One partial response (patient 7, fallopian tube cancer) and 3 disease stabilizations of ≥ 6 cycles (patients 20, 30, and 31; granulosa cell ovarian, ovarian, and endometrial cancer, respectively) occurred in this group. Patient 7 was treated with multiple regimens prior to enrollment and remained on study for 14 cycles.

### Patients with breast cancer

Nine patients with breast cancer were evaluable. Patient 33 (DL8), who took letrozole for 8 years and exemestane prior to trial enrollment, experienced a partial response; patients 3, 10, and 21 (DL1, DL3, and DL6, respectively) experienced stable disease for ≥ 6 cycles. Patient 3 died following disease progression during cycle 7. All three of these patients had previously taken both tamoxifen and anastrozole, in addition to numerous other agents.

### Hormone receptor status

Patients with breast cancer (*n* = 9) were required to have confirmed HR+ status to enroll in this study. Of those, 6 patients (patients 10, 21, 24, 27, 32, and 33) were ER+/PR+, while 3 (patients 3, 6, and 9) were ER+/PR-. The mean number of cycles completed (3.0 ± 2.6 cycles for 3 ER+/PR- patients, 6.7 ± 4.4 cycles for 6 ER+/PR+ patients) was not significantly different between these groups (*P* = 0.23). Patients with gynecologic tumors did not require determination of HR status to enroll in this study. However, there were 4 evaluable patients with ovarian cancer (patients 2, 18, 26, and 30) whose HR statuses were reported; all 4 of these patients’ tumors were ER+. Patients with ER+ ovarian cancer remained on study for 4.0 ± 4.0 cycles. This was not significantly different from patients with undetermined ER status ovarian cancer (*n* = 5, patients 1, 11, 12, 13, and 15), who remained on study for 2.0 ± 1.2 cycles (*P* = 0.32). Mean cycles completed on study did not differ significantly between evaluable patients with documented ER+ ovarian cancer or ER+ breast cancer (4.0 ± 4.0 and 5.1 ± 4.4 respectively, *P* = 0.57). Determination of HR status for patients with other gynecologic tumor histologies was not required, precluding further comparisons.

## DISCUSSION

Results from a recently published clinical trial by Goetz et al. in which Z-endoxifen was administered at doses up to 160 mg/day to women with hormone refractory metastatic breast cancer, indicated that Z-endoxifen was well tolerated and associated with clinical benefit (clinical benefit rate [CBR] defined as complete response, partial response, or stable disease for ≥ 6 cycles) [27]. Here, in this separate study, we investigated the pharmacokinetics, safety, and efficacy of Z-endoxifen using doses up to 360 mg daily in a cohort of patients composed predominantly of non-breast malignancies (31 of 40 patients had malignancies other than breast cancer).

Oral administration of Z-endoxifen in this study produced plasma levels well above those achieved with therapeutic doses of tamoxifen [26]. In women administered tamoxifen monotherapy at 20 mg/day, steady-state Z-endoxifen concentrations of > 5.97 ng/mL were associated with a 26% lower risk of a breast cancer event (recurrence or new primary breast tumors) [21]. In contrast, a prospective study of women with ER+ breast cancer treated with a short course of adjuvant tamoxifen (median 2.6 years) reported no such association [30]. However, women in this latter study were also pre-treated with chemotherapy (61%) and trastuzumab (9%) and received aromatase inhibitors after tamoxifen, thus obscuring the role of Z-endoxifen. In our study, all patients achieved Z-endoxifen plasma concentrations > 5.97 ng/mL; the mean day 28 trough concentration at DL1 was 131.7 ng/mL (range: 48.8–206.6 ng/mL, Supplementary Table 2). The average AUC values on day 28 at DL1-DL6 was 1.81- to 4.36-fold higher than the corresponding averages on day 1. Day 28 samples were not obtained for patients at DL7 or DL8. Given the average concentration of 676 ng/mL on day 1 at DL8, the estimated day 28 values would range from 1220 ng/mL to 2970 ng/mL (3.3 μM to 7.9 μM) if accumulation was similar to the lower dose levels. Of the 40 patients enrolled in the present study, lymphopenia (*n* = 11) and anemia (*n* = 10) were the most common grade ≥ 2 adverse events and one instance of grade 2 nausea was reported. In contrast, in a phase 1 study of high-dose tamoxifen (200 mg/m^2^/day) in men with hormone-refractory prostate cancer, the mean plasma tamoxifen concentration was 2.94 (± 1.15) μM [31]. In that study, the most common grade ≥ 2 adverse events were gait alterations (14 of 34 patients), nausea (6 of 34), and vomiting (4 of 34). These data suggest that high dose Z-endoxifen is not only well-tolerated, but its adverse event profile may differ from tamoxifen. However, given that patients in the current study remained on Z-endoxifen for a median of 4 cycles, additional long-term safety data are needed.

The rationale for studying Z-endoxifen in tumors other than breast cancer is based on prior data demonstrating that tamoxifen can induce complete and partial responses in 4% and 9%, respectively, of patients with ovarian cancer, while an additional 38% of patients with ovarian cancer have been reported to achieve stable disease with tamoxifen treatment [32]. Furthermore, patients with desmoid tumors have experienced complete or partial regressions with a combination of high-dose tamoxifen and sulindac [33]. Clinical benefit in the setting of tamoxifen has also been demonstrated with a wide range of other cancers [34]. Three patients in this study experienced partial responses, and 8 others achieved stable disease for at least 6 cycles, resulting in an overall CBR of 32.5%. However, the difference in CBR varied according to dose level. Specifically, the CBR was 18.8% in those treated from DL1 to DL5 (3/16 patients, doses from 20 to 140 mg daily). In contrast, the CBR was 44.4% for patients treated from DL6 to DL8 (8/18 patients, doses from 200 to 360 mg daily), suggesting a possible dose response with Z-endoxifen. This distinction is noteworthy as Z-endoxifen doses at DL6 through DL8, associated with the highest CBR reported here, were not studied in the previous Z-endoxifen trial, where the highest dose was 160 mg/day and the overall CBR was 26.3% [27]. Among the broad categories of patient’s diagnoses enrolled in this study (desmoid tumors, gynecologic malignancies, or hormone-receptor positive solid tumors), patients with desmoid tumors had the highest CBR (75%) observed in 3/4 patients. The CBR for patients with gynecologic malignancies was 20% (4/20 patients); whereas for hormone receptor-positive breast cancer, the CBR was 44% (4/9 patients).

A notable finding in this study was the observation of antitumor activity in patients with prior progression on tamoxifen, including one with partial response and five with stable disease. This included three patients with desmoid tumors who remained on study for an extended period of time (62+ cycles, 24 cycles, and 11 cycles), suggesting a benefit from Z-endoxifen for patients with desmoid tumors. In addition, five patients with breast cancer were treated on study for at least 5 cycles, including four who had previously received tamoxifen.

In this study, we performed ^18^F-FES imaging and demonstrated that FES tracer uptake could be reduced with Z-endoxifen treatment; however, the change in uptake was not predictive of clinical response [29]. These data, along with antitumor activity in patients with prior progression on tamoxifen and prior observations that desmoid tumors do not express either ERα or PR [35], suggest that the antitumor activity seen with the high dose Z-endoxifen may be through non-ER related mechanisms. Hawse and colleagues [36] analyzed gene expression changes at the RNA level in MCF7 breast cancer cells and reported differences in the number and functions of genes enhanced or suppressed in response to increasing Z-endoxifen concentration in the presence of pharmacologically relevant concentrations of estrogen (10 nM), tamoxifen (300 nM), NDM-tamoxifen (700 nM), and 4-hydroxy-tamoxifen (7 nM). Both the number of genes suppressed and number of genes induced increased with the concentration of Z-endoxifen from 20 nM to 1000 nM. Furthermore, they reported changes in gene expression of tamoxifen-treated cells following supplemental treatment with Z-endoxifen.

Additional preclinical and clinical data demonstrate that Z-endoxifen can elicit major responses in ER+ breast cancer that has progressed on tamoxifen [27, 34, 37]. Despite these data in breast cancer, the optimal dose or concentration of Z-endoxifen in other tumors (e.g., desmoid tumors) is unknown; however, our observation that high dose Z-endoxifen elicits antitumor activity in patients with non-breast malignancies would be in keeping with the data already observed demonstrating Z-endoxifen antitumor activity in breast cancers that have progressed on tamoxifen. Furthermore, the overall safety profile, achievable plasma concentrations of Z-endoxifen, and clinical efficacy seen in this trial indicate that this agent may particularly benefit patients who have progressed on tamoxifen treatment and suggest that further studies of Z-endoxifen should be considered in patients with non-breast (e.g., desmoid) malignancies.

## MATERIALS AND METHODS

### Patient selection

This study enrolled patients ≥ 18 years of age with gynecological tumors, desmoid tumors, histologically-documented HR+ (ER+/PR+, ER+/PR-, or ER-/PR+) breast cancer, or other solid tumors that were ER+ or PR+ by immunohistochemistry (any positive expression). Patients with metastatic breast cancer who had received at least one prior chemotherapy regimen for metastatic disease were eligible if they had also received prior treatment with tamoxifen and/or an aromatase inhibitor (if post-menopausal) with at least one hormonal regimen in the metastatic setting; patients with HER2+ breast cancer were eligible if their disease had progressed after at least one prior HER2-directed regimen for metastatic disease. All other patients must have progressed on at least one line of standard-of-care therapy.

Patients were required to have a life expectancy > 3 months, an Eastern Cooperative Group (ECOG) performance status ≤ 2, and adequate organ and marrow function, defined as absolute neutrophil ≥ 1,500/μL, platelets ≥ 100,000/μL, total bilirubin ≤ 1.5 × institutional upper limit of normal (ULN), AST (i.e., SGOT, aspartate aminotransferase) or ALT (i.e., SGPT, alanine aminotransferase) ≤ 2.5 × ULN, creatinine < 1.5 × ULN or creatinine clearance ≥ 60 mL/min/1.73 m^2^. Previous therapy must have been completed at least 4 weeks prior to enrollment. Patients taking concomitant medications known to be sensitive substrates of CYP450 enzymes were switched to other medications one week prior to starting therapy. Exclusion criteria included unstable or untreated brain metastasis, untreated spinal cord metastasis or metastasis close to vital organs, pregnancy, and co-morbidity with clinically significant intercurrent illnesses that could compromise participation. The trial was conducted under a National Cancer Institute (NCI)-sponsored IND with institutional review board approval at the NIH Clinical Center; informed written consent was obtained from all participants. Protocol design and conduct followed all applicable regulations, guidances, and local policies.

### Study design

This was an open-label trial of Z-endoxifen in patients with advanced solid tumors (https://clinicaltrials.gov/ identifier: NCT01273168). Z-endoxifen-HCl was supplied by the Pharmaceutical Management Branch, National Cancer Institute, as 20 and 40 mg capsules; agent was administered orally once daily on a continuous schedule in 28-day cycles with a starting dose of 20 mg/day taken either 1 hour before or 2 hours after meals. Dose escalation followed a traditional 3+3 design in which patients were dose-escalated to the next dose level (DL) in cohorts of 3 patients until dose-limiting toxicity (DLT) was observed. DLT was defined as an adverse event that occurred during cycle 1, was thought to be related to study drug administration, and met one of the following criteria: grade ≥ 3 non-hematologic toxicities (except diarrhea, nausea and vomiting, alopecia, rising creatinine or electrolyte toxicities that resolved within 24 hours, or intolerable estrogen withdrawal symptoms) or grade ≥ 3 hematologic toxicities (except neutropenia lasting less than 5 days, lymphopenia, or anemia). Eight dose levels (DL1-8) were examined: 20, 40, 60, 100, 140, 200, 280, and 360 mg, respectively. Two patients at DL8 received the free base form of Z-endoxifen on day 1 rather than the hydrochloride salt to assess the pharmacokinetics of that formulation.

### Specimen collection

A 3-mL blood sample was collected in a K2 EDTA tube on day 1 of cycle 1 before drug administration and at the following times post-first dose: 0.5, 1, 2, 3, 4, 6, 8, and 24 hours. Samples were collected at the same time points on day 28 of cycle 1 for DL1-6 until a protocol amendment eliminated the requirement for these samples at the higher dose levels. Samples were wrapped in aluminum foil after collection to protect from light exposure. A 10-mL aliquot of urine was collected before agent administration on cycle 1 day 1, for 24 hours after dosing on day 1, and before drug administration on cycle 2 day 1; samples were refrigerated prior to analysis.

### Pharmacokinetic analysis

Plasma and urine concentrations of Z- and E-endoxifen were measured using a validated, post-column fluorescence derivatization HPLC assay [38]. Non-compartmental pharmacokinetic parameters were calculated using WinNonlin version 7.0 (Pharsight Corp., Mountainview, CA, USA). A 2 μM C_max_ target was established for day 28 plasma concentration. The amount of Z-endoxifen excreted in urine was measured over a 24-hour collection period.

### PET/CT imaging

The uptake of ^18^F-FES was measured using PET/CT imaging in patients enrolled in the present trial before and after 1–5 days of treatment with oral Z-endoxifen HCl as previously described [29]. Five additional patients were scanned following the publication of the imaging approach. The images presented here are from one of these 5 patients.

### Safety and efficacy evaluations

Eye examinations were performed at baseline, every six months while on study, and if clinically indicated. CT scans were performed at baseline, and tumor response was assessed every 2 cycles (8 weeks; every 16 weeks for patients on study for more than 12 months) based on the Response Evaluation Criteria in Solid Tumors (RECIST) version 1.1 [39]. A confirmatory scan was performed after at least 4 weeks to confirm objective response.

Toxicities were graded using Common Terminology Criteria for Adverse Events (CTCAE) version 4.0. Toxicities were required to resolve to grade 2 or below prior to initiation of the next cycle. Occurrence of a DLT was to result in a dose reduction following resolution to grade ≤ 2. No more than 2 dose reductions were allowed per patient on study. The MTD was defined as the highest dose level at which no more than 1 in 6 patients experienced a DLT.

## SUPPLEMENTARY MATERIALS




