# Supplementary material for: Phase 1 study of Z-endoxifen in patients with advanced gynecologic, desmoid, and hormone receptor-positive solid tumors
Source: Oncotarget. 2021 Feb 16;12(4):268–77. doi: 10.18632/oncotarget.27887 (PMC7899551; doi:10.18632/oncotarget.27887)
Supplement: Supplementary file 1 [file oncotarget-12-268-s001.pdf]

Phase 1 study of Z-endoxifen in patients with advanced gynecologic, desmoid, and hormone receptor-positive solid tumors

SUPPLEMENTARY MATERIALS

Supplementary Table 1: Patients' past tamoxifen and/or aromatase inhibitor treatment by cancer type

Histology	Tamoxifen only	Aromatase Inhibitor only	Both	Clinical Benefit[b]
**Ovarian cancer (*n* = 9)[a]**	0	2	2	2
**Breast cancer (*n* = 9)**	0	2	7	4
**Desmoid (*n* = 4)**	2	0	0	3
**Fallopian tube cancer (*n* = 2)**	0	1	0	1
**Granulosa cell ovarian (*n* = 2)**	0	1	0	0
**Total**	2	6	9	10

[a]*n*-values indicate the total number of evaluable patients in the trial with each cancer type, not the number that had received tamoxifen and/or aromatase inhibitors prior to enrolling. [b]Clinical benefit indicates the number of patients in this trial who experienced a partial response or stable disease for at least 6 cycles by tumor type, regardless of previous tamoxifen therapy. One patient with endometrial cancer experienced clinical benefit (patient 31, stable disease for 7 cycles) but had not received prior tamoxifen or aromatase inhibitor therapy; this patient is not included in this table.

Supplementary Table 2: Pharmacokinetics by patient, days 1 and 28[a]. See Supplementary Table 2

Supplementary Table 3: Pharmacokinetics for days 1 and 2, patients 35 and 36[a]

Day	Patient	Dose (mg)	T_{max} (h)	C_{max} (ng/nL)	C24 h (ng/mL)	AUC(0–24 h) (ng/mL•h)
1	1010035	360	6.0	849	12	8959
1	1010036	360	4.0	2699	1568	41537
		**Mean:**	**5.0**	**1774**	**790**	**25248**
		**SD:**	**1.4**	**1308**	**1100**	**23036**
		**CV:**	**28**	**74**	**139**	**91**
**Day**	**Patient**	**Dose (mg)**	**T_{max} (h)**	**C_{max} (ng/nL)**	**C24 h (ng/mL)**	**AUC(0–24 h) (ng/mL•h)**
2	1010035	360	8.0	1240	827	23709
2	1010036	360	4.0	3479	2111	59421
		**Mean:**	**6.0**	**2360**	**1469**	**41565**
		**SD:**	**2.8**	**1583**	**908**	**25252**
		**CV:**	**47**	**67**	**62**	**61**

[a]These patients received the free base form of Z-endoxifen on day 1 and the HCl salt from day 2 onward.

Supplementary Table 4: All drug-related adverse events by grade and dose level[a]

		**Dose Level:**	**1**	**2**	**3**	**4**	**5**	**6**	**7**	**8**
		**Dose (mg):**	**20**	**40**	**60**	**100**	**140**	**200**	**280**	**360**
**Adverse Event**	**Grade**	***n* (patients):**	**3**	**6**	**3**	**3**	**3**	**3**	**7**	**12**
Abdominal pain	3								1	
Anemia	2			1	1	1			2	4
	3									1
Colonic perforation	4									1
Constipation	2									1
Dehydration	3									1
Diarrhea	3								1	
Dyspepsia	2									1
Elevated ALT	2			1					1	2
	3								1	1
Elevated AST	2								1	1
Fatigue	2			1			1	1	1	1
Hypertension	2							1		
Hyponatremia	3									1
Hypophosphatemia	2								1	2
	3									2
	4								1	
Leukopenia	2				1		1			1
Lymphopenia	2			3		1			1	5
	3									1
Mucositis (oral)	2			1						
Nausea	2									1
Neutropenia	2				1		1			
Rash	2									1
Reflux	2						1			
Stroke	2									1
Thromboembolic event	2			1						1
	4			1						
Vestibular disorder	2			1						

[a]All adverse events graded ≥ 2 and determined to be possibly, probably, or definitely related to study drug for each patient. Some patients experienced multiple adverse events and all that were at least grade 2 are included. Abbreviations: ALT, alanine aminotransferase; AST, aspartate aminotransferase.
