# Supplementary material for: Phase 1 study of Z-endoxifen in patients with advanced gynecologic, desmoid, and hormone receptor-positive solid tumors
Source: Oncotarget. 2021 Feb 16;12(4):268–77. doi: 10.18632/oncotarget.27887 (PMC7899551; doi:10.18632/oncotarget.27887)
Supplement: Supplementary file 2 [file oncotarget-12-268-s002.docx]

Supplementary Table 2: Pharmacokinetics by patient, days 1 and 28^a^

| **DL 1** |  |  |  |  |  |  |  |  |
| --- | --- | --- | --- | --- | --- | --- | --- | --- |
| Patient | Dose (mg) | Day | T_max_ (h) | C_max_ (ng/mL) | C24h (ng/mL) | AUC_(0-24h)_ (ng/mL·h) | Accumulation (AUC Day 28/ AUC Day 1) | Adjusted Half-Life (h) |
| 1010001 | 20 | 1 | 3.0 | 76.9 | 25.2 | 1030 |  |  |
| 1010002 | 20 | 1 | 4.0 | 61.2 | 32.6 | 1055 |  |  |
| 1010003 | 20 | 1 | 4.0 | 67.7 | 32.7 | 1169 |  |  |
|  | **Mean:** |  | **3.7** | **68.6** | **30.2** | **1084** |  |  |
|  | **SD:** |  | **0.6** | **7.9** | **4.3** | **74** |  |  |
|  | **CV:** |  | **16** | **11** | **14** | **7** |  |  |
|  |  |  |  |  |  |  |  |  |
| Patient | Dose (mg) | Day | T_max_ (h) | C_max_ (ng/mL) | C24h (ng/mL) | AUC_(0-24h)_ (ng/mL·h) | Accumulation (AUC Day 28/ AUC Day 1) | Adjusted Half-Life (h) |
| 1010001 | 20 | 28 | 0.0 | 200.6 | 48.8 | 2959 | 2.87 | 38.9 |
| 1010002 | 20 | 28 | 1.0 | 283.7 | 139.6 | 4469 | 4.24 | 61.8 |
| 1010003 | 20 | 28 | 1.0 | 374.1 | 206.6 | 5313 | 4.55 | 67.0 |
|  | **Mean:** |  | **0.7** | **286.1** | **131.7** | **4247** | **3.9** | **55.9** |
|  | **SD:** |  | **0.6** | **86.8** | **79.2** | **1192** | **0.9** | **15.0** |
|  | **CV:** |  | **87** | **30** | **60** | **28** | **23** | **27** |
|  |  |  |  |  |  |  |  |  |
| **DL 2** |  |  |  |  |  |  |  |  |
| Patient | Dose (mg) | Day | T_max_ (h) | C_max_ (ng/mL) | C24h (ng/mL) | AUC_(0-24h)_ (ng/mL·h) | Accumulation (AUC Day 28/ AUC Day 1) | Adjusted Half-Life (h) |
| 1010004 | 40 | 1 | 4.0 | 83.6 | 43.6 | 1290 |  |  |
| 1010006 | 40 | 1 | 4.0 | 121.1 | 54.9 | 1715 |  |  |
| 1010007 | 40 | 1 | 4.0 | 93.8 | 33.7 | 1336 |  |  |
| 1010008 | 40 | 1 | 4.0 | 102.3 | 55.2 | 1764 |  |  |
| 1010009 | 40 | 1 | 4.0 | 160.3 | 75 | 2162 |  |  |
|  | **Mean:** |  | **4.0** | **112.2** | **52.5** | **1653** |  |  |
|  | **SD:** |  | **0.0** | **30.2** | **15.4** | **356** |  |  |
|  | **CV:** |  | **0** | **27** | **29** | **22** |  |  |

| Patient | Dose (mg) | Day | T_max_ (h) | C_max_ (ng/mL) | C24h (ng/mL) | AUC_(0-24h)_ (ng/mL·h) | Accumulation (AUC Day 28/ AUC Day 1) | Adjusted Half-Life (h) |
| --- | --- | --- | --- | --- | --- | --- | --- | --- |
| 1010006 | 40 | 28 | 3.0 | 321.1 | 135.5 | 5567 | 3.25 | 45.2 |
| 1010007 | 40 | 28 | 1.0 | 231.8 | 111.8 | 3871 | 2.90 | 39.3 |
| 1010008 | 40 | 28 | 6.0 | 220.7 | 194.5 | 4832 | 2.74 | 36.6 |
|  | **Mean:** |  | **3.3** | **257.9** | **147.3** | **4756** | **3.0** | **40.4** |
|  | **SD:** |  | **2.5** | **55.0** | **42.6** | **850** | **0.3** | **4.4** |
|  | **CV:** |  | **75** | **21** | **29** | **18** | **9** | **11** |
|  |  |  |  |  |  |  |  |  |
| **DL 3** |  |  |  |  |  |  |  |  |
| Patient | Dose (mg) | Day | T_max_ (h) | C_max_ (ng/mL) | C24h (ng/mL) | AUC_(0-24h)_ (ng/mL·h) | Accumulation (AUC Day 28/ AUC Day 1) | Adjusted Half-Life (h) |
| 1010010 | 60 | 1 | 24.0 | 89.7 | 89.7 | 1622 |  |  |
| 1010011 | 60 | 1 | 6.0 | 109.0 | 72.2 | 1953 |  |  |
| 1010012 | 60 | 1 | 6.1 | 166.0 | 86.2 | 2836 |  |  |
|  | **Mean:** |  | **12.0** | **121.6** | **82.7** | **2137** |  |  |
|  | **SD:** |  | **10.4** | **39.7** | **9.3** | **628** |  |  |
|  | **CV:** |  | **86** | **33** | **11** | **29** |  |  |
|  |  |  |  |  |  |  |  |  |
| Patient | Dose (mg) | Day | T_max_ (h) | C_max_ (ng/mL) | C24h (ng/mL) | AUC_(0-24h)_ (ng/mL·h) | Accumulation (AUC Day 28/ AUC Day 1) | Adjusted Half-Life (h) |
| 1010010 | 60 | 28 | 0.5 | 199.6 | 169.4 | 3432 | 2.12 | 26.0 |
| 1010011 | 60 | 28 | 24.0 | 521.2 | 521.2 | 9961 | 5.10 | 76.2 |
| 1010012 | 60 | 28 | 8.0 | 531.1 | 345.6 | 10846 | 3.82 | 54.9 |
|  | **Mean:** |  | **10.8** | **417.3** | **345.4** | **8080** | **3.7** | **52.4** |
|  | **SD:** |  | **12.0** | **188.6** | **175.9** | **4049** | **1.5** | **25.2** |
|  | **CV:** |  | **111** | **45** | **51** | **50** | **41** | **48** |

|  |  |  |  |  |  |  |  |  |
| --- | --- | --- | --- | --- | --- | --- | --- | --- |
| **DL 4** |  |  |  |  |  |  |  |  |
| Patient | Dose (mg) | Day | T_max_ (h) | C_max_ (ng/mL) | C24h (ng/mL) | AUC_(0-24h)_ (ng/mL·h) | Accumulation (AUC Day 28/ AUC Day 1) | Adjusted Half-Life (h) |
| 1010013 | 100 | 1 | 6.0 | 264 | 133 | 4641 |  |  |
| 1010014 | 100 | 1 | 6.0 | 301 | 157 | 4834 |  |  |
| 1010015 | 100 | 1 | 1.0 | 324 | 175 | 5511 |  |  |
|  | **Mean:** |  | **4.3** | **296** | **155** | **4995** |  |  |
|  | **SD:** |  | **2.9** | **30** | **21** | **457** |  |  |
|  | **CV:** |  | **67** | **10** | **14** | **9** |  |  |
|  |  |  |  |  |  |  |  |  |
| Patient | Dose (mg) | Day | T_max_ (h) | C_max_ (ng/mL) | C24h (ng/mL) | AUC_(0-24h)_ (ng/mL·h) | Accumulation (AUC Day 28/ AUC Day 1) | Adjusted Half-Life (h) |
| 1010013 | 100 | 28 | 2.0 | 1301 | ND | ND | ND | ND |
| 1010014 | 100 | 28 | 4.0 | 719 | 428 | 13286 | 2.75 | 36.8 |
| 1010015 | 100 | 28 | ND | ND | ND | ND | ND | ND |
|  | **Mean:** |  | **3.0** | **1010** | **428.0** | **13286** | **2.7** | **36.8** |
|  | **SD:** |  | **1.4** | **411.5** | **N/A** | **N/A** | **N/A** | **N/A** |
|  | **CV:** |  | **47** | **41** | **N/A** | **N/A** | **N/A** | **N/A** |
|  |  |  |  |  |  |  |  |  |
| **DL 5** |  |  |  |  |  |  |  |  |
| Patient | Dose (mg) | Day | T_max_ (h) | C_max_ (ng/mL) | C24h (ng/mL) | AUC_(0-24h)_ (ng/mL·h) | Accumulation (AUC Day 28/ AUC Day 1) | Adjusted Half-Life (h) |
| 1010016 | 140 | 1 | 3.0 | 380 | 209 | 7200 |  |  |
| 1010017 | 140 | 1 | 6.0 | 957 | 509 | 14650 |  |  |
| 1010018 | 140 | 1 | 4.0 | 494 | 216 | 7425 |  |  |
|  | **Mean:** |  | **4.3** | **610** | **311** | **9758** |  |  |
|  | **SD:** |  | **1.5** | **306** | **171** | **4238** |  |  |
|  | **CV:** |  | **35** | **50** | **55** | **43** |  |  |

| Patient | Dose (mg) | Day | T_max_ (h) | C_max_ (ng/mL) | C24h (ng/mL) | AUC_(0-24h)_ (ng/mL·h) | Accumulation (AUC Day 28/ AUC Day 1) | Adjusted Half-Life (h) |
| --- | --- | --- | --- | --- | --- | --- | --- | --- |
| 1010016 | 140 | 28 | 3.0 | 973 | 598 | 18211 | 2.53 | 33.1 |
| 1010017 | 140 | 28 | 1.0 | 1485 | ND | ND | ND | ND |
| 1010018 | 140 | 28 | 2.0 | 881 | 528 | 16591 | 2.23 | 28.1 |
|  | **Mean:** |  | **2.0** | **1113** | **563** | **17401** | **2.4** | **30.6** |
|  | **SD:** |  | **1.0** | **325** | **49** | **1146** | **0.2** | **3.5** |
|  | **CV:** |  | **50** | **29** | **9** | **7** | **9** | **12** |
|  |  |  |  |  |  |  |  |  |
| **DL 6** |  |  |  |  |  |  |  |  |
| Patient | Dose (mg) | Day | T_max_ (h) | C_max_ (ng/mL) | C24h (ng/mL) | AUC_(0-24h)_ (ng/mL·h) | Accumulation (AUC Day 28/ AUC Day 1) | Adjusted Half-Life (h) |
| 1010019 | 200 | 1 | 8.0 | 299 | 279 | 6337 |  |  |
| 1010020 | 200 | 1 | 2.0 | 760 | 457 | 12520 |  |  |
| 1010021 | 200 | 1 | 2.0 | 492 | 271 | 8804 |  |  |
|  | **Mean:** |  | **4.0** | **517** | **336** | **9220** |  |  |
|  | **SD:** |  | **3.5** | **232** | **105** | **3112** |  |  |
|  | **CV:** |  | **87** | **45** | **31** | **34** |  |  |
|  |  |  |  |  |  |  |  |  |
| Patient | Dose (mg) | Day | T_max_ (h) | C_max_ (ng/mL) | C24h (ng/mL) | AUC_(0-24h)_ (ng/mL·h) | Accumulation (AUC Day 28/ AUC Day 1) | Adjusted Half-Life (h) |
| 1010019 | 200 | 28 | 2.0 | 984 | 787 | 16690 | 2.63 | 34.8 |
| 1010020 | 200 | 28 | 2.0 | 2574 | 1814 | 52925 | 4.23 | 61.6 |
| 1010021 | 200 | 28 | 2.0 | 1364 | 606 | 22641 | 2.57 | 33.8 |
|  | **Mean:** |  | **2.0** | **1641** | **1069** | **30752** | **3.1** | **43.4** |
|  | **SD:** |  | **0.0** | **830** | **652** | **19432** | **0.9** | **15.8** |
|  | **CV:** |  | **0** | **51** | **61** | **63** | **30** | **36** |

| **DL 7** |  |  |  |  |  |  |  |  |
| --- | --- | --- | --- | --- | --- | --- | --- | --- |
| Patient | Dose (mg) | Day | T_max_ (h) | C_max_ (ng/mL) | C24h (ng/mL) | AUC_(0-24h)_ (ng/mL·h) |  |  |
| 1010022 | 280 | 1 | 2.0 | 825 | 345 | 10460 |  |  |
| 1010023 | 280 | 1 | 3.0 | 715 | 384 | 11355 |  |  |
| 1010024 | 280 | 1 | 2.0 | 887 | 467 | 13153 |  |  |
| 1010025 | 280 | 1 | 4.0 | 796 | 337 | 13081 |  |  |
| 1010026 | 280 | 1 | 3.0 | 1395 | 687 | 24217 |  |  |
| 1010027 | 280 | 1 | 4.0 | 1007 | 574 | 16532 |  |  |
| 1010028 | 280 | 1 | 3.0 | 888 | 485 | 15935 |  |  |
|  | **Mean:** |  | **3.0** | **930** | **468** | **14962** |  |  |
|  | **SD:** |  | **0.8** | **224** | **128** | **4640** |  |  |
|  | **CV:** |  | **27** | **24** | **27** | **31** |  |  |
|  |  |  |  |  |  |  |  |  |
| **DL 8** |  |  |  |  |  |  |  |  |
| Patient | Dose (mg) | Day | T_max_ (h) | C_max_ (ng/mL) | C24h (ng/mL) | AUC_(0-24h)_ (ng/mL·h) |  |  |
| 1010029 | 360 | 1 | 6.0 | 1841 | 1190 | 32079 |  |  |
| 1010030 | 360 | 1 | 2.0 | 1352 | 722 | 21926 |  |  |
| 1010031 | 360 | 1 | 3.0 | 1374 | 653 | 23576 |  |  |
| 1010032 | 360 | 1 | 6.0 | 1432 | 801 | 20747 |  |  |
| 1010033 | 360 | 1 | 4.0 | 1437 | 658 | 22520 |  |  |
| 1010034 | 360 | 1 | 3.0 | 780 | 213 | 9274 |  |  |
| 1010037 | 360 | 1 | 3.0 | 948 | 493 | 13704 |  |  |
|  | **Mean:** |  | **3.9** | **1309** | **676** | **20547** |  |  |
|  | **SD:** |  | **1.6** | **349** | **297** | **7325** |  |  |
|  | **CV:** |  | **41** | **27** | **44** | **36** |  |  |

^a^ Data include all samples collected and analyzed for patients receiving Z-endoxifen HCl. Patients 35 and 36 received the free base form of Z-endoxifen on day 1 and their data are reported in Supplementary Table 3.
